# The causes of species richness patterns among clades

**DOI:** 10.1098/rspb.2023.2436

**Published:** 2024-01-24

**Authors:** Dan Yu, John J. Wiens

**Affiliations:** ^1^ The Key Laboratory of Aquatic Biodiversity and Conservation of Chinese Academy of Science, Institute of Hydrobiology, Chinese Academy of Sciences, Wuhan, Hubei 430072, People's Republic of China; ^2^ Department of Ecology and Evolutionary Biology, University of Arizona, Tucson, AZ 85721-0088, USA

**Keywords:** clade age, diversification, macroevolution, phylogeny, species richness

## Abstract

Two major types of species richness patterns are spatial (e.g. the latitudinal diversity gradient) and clade-based (e.g. the dominance of angiosperms among plants). Studies have debated whether clade-based richness patterns are explained primarily by larger clades having faster rates of species accumulation (speciation minus extinction over time; diversification-rate hypothesis) or by simply being older (clade-age hypothesis). However, these studies typically compared named clades of the same taxonomic rank, such as phyla and families. This study design is potentially biased against the clade-age hypothesis, since clades of the same rank may be more similar in age than randomly selected clades. Here, we analyse the causes of clade-based richness patterns across the tree of life using a large-scale, time-calibrated, species-level phylogeny and random sampling of clades. We find that within major groups of organisms (animals, plants, fungi, bacteria, archaeans), richness patterns are most strongly related to clade age. Nevertheless, weaker relationships with diversification rates are present in animals and plants. These overall results contrast with similar large-scale analyses across life based on named clades, which showed little effect of clade age on richness. More broadly, these results help support the overall importance of time for explaining diverse types of species richness patterns.

## 1. Introduction

Understanding the origins of species richness patterns is an important goal of both ecology and evolutionary biology. Two main types of species richness patterns have been studied most frequently: spatial and clade-based. Spatial patterns include the latitudinal diversity gradient, elevational richness patterns and the higher richness of terrestrial habitats relative to marine habitats. Clade-based richness patterns are far less studied, but no less dramatic. For example, despite the existence of approximately 34 animal phyla, 80% of extant, described animal species belong to just one phylum (Arthropoda [1]).

Two main, non-exclusive hypotheses have been proposed to directly explain these patterns of species richness among clades (e.g. [2,3]). First, clades may have more species because they diversify more rapidly (diversification-rate hypothesis), leading to a larger number of species in a given span of time. Diversification rates reflect the balance of both speciation and extinction rates over time [4]. Second, clades may have more species simply because they are older and have had more time to accumulate species than younger clades (clade-age hypothesis).

The general causes of clade-based richness patterns have not been widely studied, but the few broad-scale studies that have addressed these patterns have often arrived at divergent conclusions. For example, McPeek & Brown [2] analysed 163 species-level phylogenies from arthropods, chordates and molluscs. They also analysed data on richness and ages from higher taxa (e.g. orders) from vertebrates and insects. For both datasets, they found positive correlations between clade age and species richness but no significant correlations between diversification rates and richness. Rabosky *et al*. [5] analysed families of animals, plants and fungi. They concluded that richness patterns were not explained by clade age. They also concluded that they were not explained by variation in diversification rates, but did not directly test this hypothesis. Hedges *et al*. [6] examined a large-scale, species-level phylogeny including many eukaryote groups. They inferred that diversification rates were mostly constant among clades, and that differences in richness among clades were therefore caused by differences in clade ages. However, they did not test for relationships between richness and diversification rates of clades. They did analyse relationships between richness and clade ages, but only among families and genera of mammals and birds. In this case, they found significant (but relatively weak) relationships when using crown-group ages (age of the oldest split among extant lineages within a clade) but not using stem-group ages (the time when the clade first splits from its sister group). Furthermore, subsequent analyses found that diversification rates explained most (50–66%) variation in richness among these bird and mammal families [3]. Scholl & Wiens [7] analysed named higher-level clades across the tree of life including families, orders, classes, phyla and kingdoms. They found that richness patterns among clades of the same rank were generally explained by diversification rates and not clade ages, both across life and within major groups (i.e. animals, fungi, plants, major protist groups, archaeans, bacteria). In summary, these studies often arrived at divergent conclusions about the causes of richness patterns among clades, but many studies did not test both of the relevant hypotheses.

**Figure 1.**
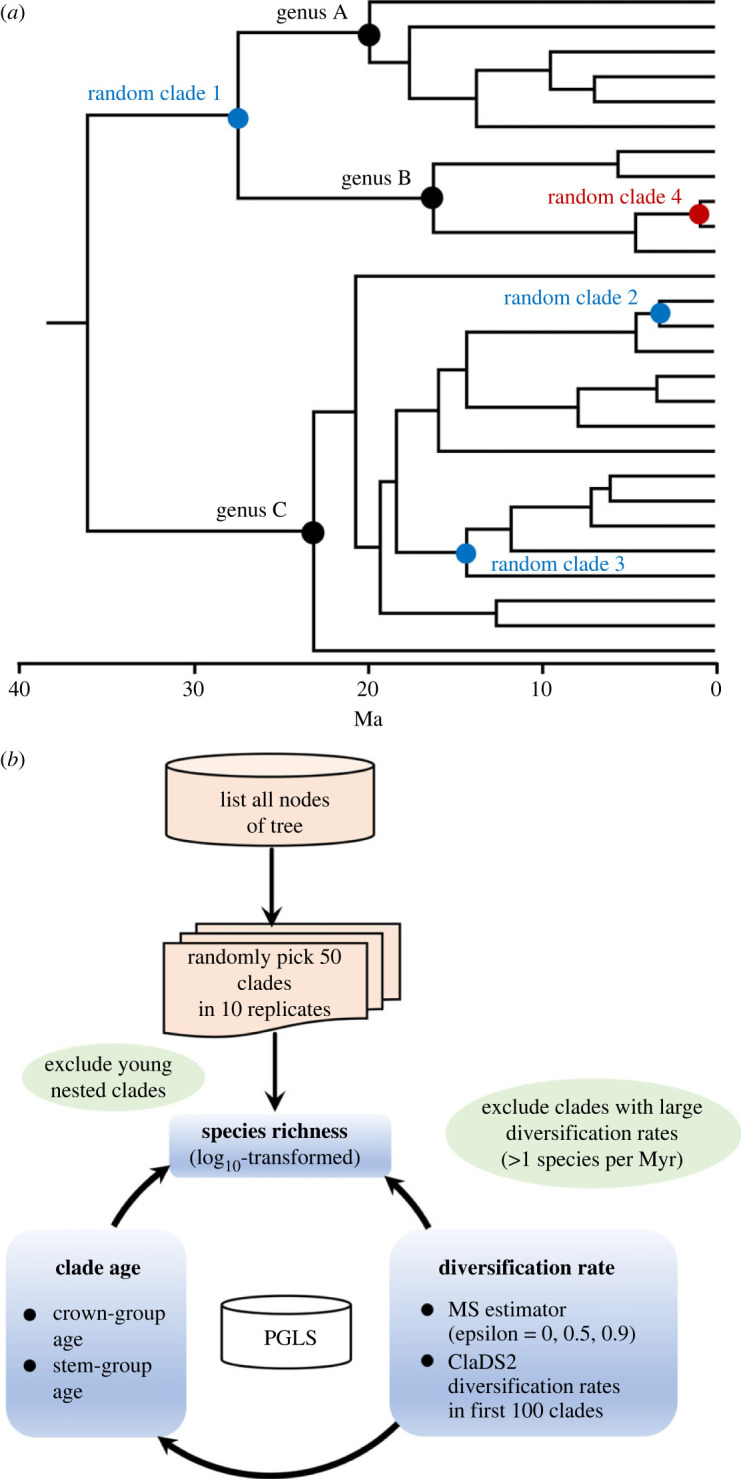
Hypothetical example and workflow for selecting clades and analysing the relationships among variables. (*a*) A time-calibrated phylogeny for a hypothetical group of organisms, illustrating the consequences of using named clades as opposed to randomly selected clades. If clades are randomly selected, they can be of almost any age. Here, the four randomly selected clades range in age from 26 to 2 million years old. By contrast, clades of the same taxonomic rank may be constrained to be of similar ages, or older ages. Here the three genera range in age from 17 to 23 million years old. Note that random clade 4 is highlighted in red because it is nested inside random clade 1. We excluded such nested clades. (*b*) Illustration of the overall workflow used in this study. For a given group of organisms (e.g. animals, plants, bacteria), we obtained a time-calibrated, species-level phylogeny. We then generated a list of all the nodes in that tree. We next randomly selected 50 clades. Clades that were nested inside of other selected clades were deleted, as were those that were extremely young (e.g. zero-length branches, and when different species in the clade had almost identical sequences). We then estimated clade ages and diversification rates for each clade. Clades with very high diversification rates (greater than 1 species per million years) were also excluded. Finally, we used phylogenetic generalized least-squares regression (PGLS) to test whether species richness (dependent variable) was related to clade age or to diversification (independent variables) among these 50 clades, testing the clade-age and diversification rate hypotheses. We also tested whether diversification rates were related to clade age. This overall procedure was repeated 10 times (each with a different random selection of 50 clades) for each major group of organisms.

These studies came to disparate conclusions, but many shared a potentially important source of bias (figure 1*a*). Specifically, most studies compared clades of the same taxonomic rank (i.e. families to families, orders to orders). Although such comparisons are intrinsically interesting, clades based on named taxa of the same rank might be more similar in age than a randomly selected set of clades [3]. Reducing the variation in clade ages may therefore bias the results against the clade-age hypothesis and in favour of the diversification-rate hypothesis. In the most extreme case, if all clades are roughly the same age, then the clade-age hypothesis cannot be supported, and all variation in richness must be explained by differences in diversification rates. At the same time, it is unclear whether the clade-age hypothesis would be supported if clades were chosen randomly, regardless of taxonomic ranks. Instead, variation in diversification rates might still drive clade-based richness patterns, or at least greatly reduce the effects of clade age on richness. Furthermore, the effect of random sampling of clades (versus comparison of named clades) on clade ages remains untested.

Here, we test the causes of clade-based richness patterns without this potential source of bias. We use a time-calibrated tree that spans all major clades of living organisms (i.e. bacteria, archaeans, plants, fungi, animals) but also include species-level relationships [8]. We randomly select clades from this tree, allowing the chosen clades to be of any age (figure 1*a,b*). We estimate the species richness, age, and diversification rate of each clade. We estimate diversification rates using two very different estimators (method-of-moments MS estimator [9] and ClaDS2 [10]), but which yield strongly related estimates. We then test whether patterns of species richness among clades are more strongly related to clade ages or diversification rates, primarily using phylogenetic generalized least-squares regression (PGLS) [11].

## 2. Material and methods

### (a) Phylogenetic information

We used the TimeTree of Life (http://www.timetree.org/) to obtain the most recent and comprehensive time-calibrated species-level trees for major clades across the Tree of Life. The fifth edition of the TimeTree of Life resource (TToL5) contains divergence-time information on 137 306 species from 4075 articles published since 1985 [8]. We used this tree to randomly sample clades within Animalia (67 685 species in tree), Plantae (51 569 species), Fungi (4795 species), Bacteria (11 074 species), Archaea (426 species) and across all of life (137 281 species). Plantae here refers to land plants (Embryophyta) and excludes green algae. Because protists do not form a monophyletic group, we did not analyse them separately, but they are potentially included in the analyses across life (depending on the random sampling of clades).

Within each major group, this tree includes species-level relationships within genera as well as relationships among genera, families, phyla and other higher taxa. However, it is not complete nor systematic in its sampling. We address the effects of incomplete species sampling in the final section of the methods below. There were also polytomies and zero-length branches in some parts of the tree. We address these issues below.

### (b) Selecting clades

We used the tree to analyse pairwise relationships between species richness and clade age, species richness and diversification rate and diversification rate and clade age (figure 1*b*). For each major group (animals, plants, fungi, bacteria, archaeans, life), we randomly selected 50 clades and then estimated their age, species richness, and diversification rate. To ensure that our results did not depend strongly on the particular selection of clades, we repeated the random selection of 50 clades 10 times for each group. We then summarized the results among these 10 replicates.

We acknowledge that both numbers (50 and 10) are somewhat arbitrary. In each replicate, we did not want to include every single clade in a given group (e.g. plants), since most sampled clades would then be nested inside of other sampled clades (figure 1*a*). This nesting would favour the clade-age hypothesis: younger clades nested inside older clades must have lower richness than the larger, older clades to which they belong. Using sets of 50 clades allowed us to select non-overlapping, non-nested clades.

To randomly select clades for each group and each replicate, we generated a numbered list of tips and nodes for the tree for each group using the function ‘get_clade_list’ in the R package *castor* [12]. We then used the numbered list of nodes as the list of clades. After running ‘get_clade_list,’ we also obtained a numbered list of parent nodes to get the stem-group age for each clade. We then used the function ‘sample’ in the R package *base* [13] to randomly pick 50 clades from the above list of clades for each group.

For the analyses across life, we randomly selected 45 eukaryote clades, four bacterial clades, and one archaean clade for each replicate. This ensured that every tree included all three domains of life, with domains sampled in rough proportion to their described species richness in the tree (although actual bacterial richness may be much higher; e.g. [14,15]). We acknowledge that archaeans may not be monophyletic (e.g. [16,17]), but our results apply to those archaeans included in our tree.

In general, most randomly selected clades were not nested within other randomly selected clades. For example, in figure 1*a*, if clade 4 was in genus B, whereas clade 1 included genus A and genus B, then clade 4 was nested in clade 1. We did not observe this problem in most major groups (animals, plants, fungi, bacteria, life). However, this problem occurred in 17/500 archaean clades, probably because of the low overall richness of archaeans. We excluded these nested clades in our regression analyses. Specifically, when one clade was nested inside another, we arbitrarily excluded the younger clade.

### (c) Age and species richness of clades

The age of a clade can be based on its crown-group age (age of the oldest split among extant lineages within a clade) or stem-group age (when the clade first splits from its sister group). Both have been used in previous studies on the relationship between clade age and richness (e.g. crown: [2,6]; stem: [5,7]). Here, we initially used crown-group ages. We also addressed whether the conclusions would be changed by using stem-group ages instead.

We estimated the age and richness of each clade as follows. We extracted the crown-group age and stem-group age of each clade in the tree for each group using the function ‘branching.times’ in the R package *ape* [18]. We also obtained a list of species in each clade with the function ‘tips’ in the R package *geiger* [19]. We counted these species to obtain the clade's richness using the function ‘length’ in the R package *base* [13]. We note that taxon sampling is incomplete for all groups, such that the number of species in the tree for a given clade may be less than the number present if the tree were fully complete. Again, we performed analyses to address this issue in the final section of the methods.

### (d) Estimating diversification rates

We used two approaches to estimate the diversification rates. As a first approach, we estimated the diversification rate of each clade using the crown-group method-of-moments estimator [9], implemented using the function ‘bd.ms’ in the R package *geiger* [19]. The MS estimator was also used in previous studies of this topic [2,7]. Simulations show strong relationships between true and estimated rates among clades for this method, including simulations in which diversification rates are faster in younger clades [20], diversification rates differ between subclades within each clade [21], and when speciation, extinction, and diversification rates vary strongly over time within each clade [22]. Therefore, this approach does not require constant rates within or between clades to accurately reflect the true diversification rates.

We note that it is not circular to use this method to assess the relationship between diversification rates and species richness, even though richness is used to estimate diversification rates. First, there is no ‘circularity’, following standard definitions of the term. Circularity would involve, for example, using a method that guarantees an outcome, then using that outcome to justify the choice of method. However, one might speculate that using species richness to calculate diversification rates makes a strong relationship between these variables inevitable. Yet, our results clearly show that such a relationship is not inevitable (nor even the most common outcome). Second, we show that an alternative method, which is not based directly on richness, gave rate estimates that were strongly related to those from the MS estimator. Third, we think that the most important consideration in choosing an estimator is whether the rate estimates are correlated with the true rates. This has been established for the MS estimator based on simulations (see above).

As usually applied, the MS estimator assumes a ratio of extinction to speciation rates (*ε*) to estimate diversification rates, rather than attempting to separately estimate and disentangle speciation and extinction rates. Following standard practice, we assumed three values of *ε* (zero, intermediate, and high extinction relative to speciation: 0, 0.5, and 0.9). We generally present results using an intermediate value (0.5). However, simulations suggest that for the crown-group estimator, an *ε* of 0.90 may generally give the most accurate results when extinction and speciation rates are highly variable among clades [21,22]. Therefore, we present these results as well. Because the MS estimator does not estimate separate speciation and extinction rates, it should not suffer from a lack of identifiability of these separate rates [23]. Most importantly, this method appears to yield strong correlations between true and estimated diversification rates [20–22], regardless of identifiability.

Some estimated diversification rates were extremely large (greater than 100 species per million years; electronic supplementary material, dataset S1) especially in archaeans, bacteria and fungi. These high rates were associated with very young clade ages, generally with just two species. There are at least four potential explanations for these very young clade ages. One possibility is that these apparently young species were included as parts of polytomies, rather than based on actual branch lengths. Another possibility is that these were misidentified or mislabelled species, such that the original studies treated the sequences as coming from two different species, but in reality the sequences were nearly identical (leading to a very young inferred divergence time between them). Third, they might reflect introgression, again leading to sequences from two different species appearing as almost identical and thus having a very short divergence time. Fourth, the two species might be phenotypically distinct but genetically indistinguishable (i.e. problematic taxonomy).

We found that this problem of very young clades occurred in all groups. In fungi, 43 out of 500 clades were less than 1 million year (Myr) old, but only three of these clades were in polytomies. In bacteria and archaeans, 105 and 62 of 500 clades each were less than 1 Myr, and 34 and 21 involved polytomies. In animals, 18/500 clades were less than 1 Myr, and none involved polytomies. In plants, 46/500 clades were less than 1 Myr, and six involved polytomies. Across life, 39/500 clades were less than 1 Myr, and four involved polytomies. Unfortunately, we did not have an obvious solution for these issues. Therefore, we excluded from the main analyses any clades with extremely large diversification rates (greater than 1 species per Myr) and clades that were in polytomies. We also present inferences with all clades included, and these yielded similar findings overall (see Results).

As a second approach to estimating diversification rates, we used the method ClaDS [24]. This Bayesian method allows speciation (*λ*) and extinction (*μ*) rates to vary while maintaining constant turnover (*ε*, where turnover is the extinction rate divided by the speciation rate, *ε* = *μ*/*λ*). ClaDS incorporates rate heterogeneity by modelling small shifts in rates at speciation events. At each split, the two new lineages are assumed to inherit new speciation rates that are sampled from a lognormal distribution. The distribution has an expected mean value of log(*αλ*) and a standard deviation of *σ*, where *λ* represents the ancestral speciation rate, *α* is a deterministic trend parameter and *σ* controls the stochasticity of rate inheritance.

We implemented ClaDS2 in the Julia package PANDA (https://github.com/hmorlon/PANDA.jl), which runs faster and more efficiently than the initial implementation of ClaDS [10]. We also refer to this method as ‘ClaDS’. We ran three Markov chain Monte Carlo (MCMC) replicates and generated the Gelman statistic [25] for the four hyperparameters (*α*, *σ*, *ε* and starting speciation rate *λ*_0_). ClaDS2 stops when the Gelman statistic is below 1.05 for all parameters. We also estimated lineage-specific speciation rates (*λ*_i_) for each branch and tip speciation rates (*λ*_tip_) for each tip.

The ClaDS2 model is significantly faster [10], but does have some limitations. First, it cannot accommodate polytomies or zero-length branches. That is because it may become stuck if a tree contains very small branches followed by long ones. Unfortunately, all trees for all groups used here contained many branches with very small values (0.0). Therefore, we did not attempt to estimate lineage-specific diversification rates based on the full tree for each group.

Instead, we conducted separate ClaDS2 analyses of the first 100 clades within each group. To avoid program termination, we replaced branches with a length of 0 with a very small value. Specifically, we used 1 million years, as recommended by one of the developers of ClaDS2 (O. Maliet 2023, personal communication). ClaDS2 analyses would otherwise not be possible. Although this made the trees no longer ultrametric, the addition of 1 million years to some branches was a small change relative to the total tree depth. We then used the function ‘get_subtrees_at_nodes' in *castor* to obtain the species-level trees within these 100 clades. However, based on our initial analyses, ClaDS2 required trees with at least four species. Therefore, clades consisting of only two or three species were excluded.

We ran the analyses for the rest of the clades with greater than 3 species in each group. Although no zero-length branches remained within each clade, some clades still had many branches with extremely short lengths followed by relatively long ones. These cases still caused difficulties for convergence (O. Maliet 2023, personal communication). Therefore, we excluded these clades entirely. Specifically we excluded clades when convergence was still not achieved after greater than 12 h. Since most clades had relatively few species, 12 h should have been more than sufficient. Additionally, we excluded all clades with extremely large diversification rates (greater than 1 species per Myr) from the MS estimator, for the reasons discussed above. We assumed that the analysed clades in each group were perfectly sampled, with the sampling fraction set by default for each clade.

For each clade, we generated a result file from the ClaDS2 analyses that could be manipulated in R [13]. This file contained the clade's epsilon (*ε*), branch-specific speciation rates (*λ*_i_) and tip speciation rates (*λ*_tip_). We then used R to read these three variables from each file.

Given that *ε* = *μ*/*λ*, we used the mean branch-specific speciation rates (*λ*_imean_) for each clade and its estimated clade-wide *ε* value to get that clade's mean branch-specific diversification rate using the equation: diversification rate = (1 − ε) * *λ*_imean_. Thus, for most analyses we used the mean diversification rate among all the branches (internal and terminal) among the sampled species in a clade. We also obtained mean speciation rates among all the branches (*λ*_imean_) and rates among only the tips (*λ*_tipmean_). Both gave broadly similar results to those using diversification rates. After removing clades with very high rates and convergence issues (see above), we obtained rate estimates for 29–49 clades for each of the six major groups (electronic supplementary material, dataset S1).

Several authors have noted a tendency for estimated diversification rates to be faster in younger clades [2,7,20,26,27]. The causes of this pattern remain unclear, with some studies claiming that the pattern is real [26] and others claiming it is artefactual [27]. Our goal was not to address this controversy. Nevertheless, we tested whether this pattern was present in our data, since previous simulation and empirical analyses [7,20] suggest that faster rates in younger clades can decouple diversification rates and richness (e.g. very young clades can have fast diversification rates but limited richness).

### (e) Statistical analyses

We first tested the relationship between species richness (dependent variable) and clade age (independent variable). We initially used ordinary least-squares regression (OLS) regression in R (version 4.2.2, [13]), since the ages and richness of tip clades cannot be phylogenetically inherited. We also used phylogenetic generalized least-squares regression (PGLS) in the R package *caper* [28], which gave similar results. We primarily focus on the PGLS results.

For the PGLS analyses, we needed to make reduced trees with one tip per clade. We first used the ‘get_subtrees_at_nodes' function in *castor* to generate a tree of 50 clades with each tip representing one of the randomly selected clades in each replicate. One species was arbitrarily selected from each clade to represent that clade (the choice of which species should not matter in a time-calibrated tree: all yield the same branch length). For the PGLS analyses, *δ* and *κ* were set to 1 while the maximum-likelihood value of *λ* was estimated for each analysis (following default settings in *caper* and standard practice). These reduced trees are available in electronic supplementary material, dataset S2.

We separately analysed the relationships between species richness (dependent variable) and diversification rate (independent variable). We also analysed the relationship between diversification rate (dependent variable) and clade age (independent variable). To improve linearity in the regression analyses, species richness was log10-transformed in all analyses. We performed limited multiple regression analyses (table 1) because typically only one predictor variable was significant (i.e. age, diversification), and the two predictor variables were generally not significantly related to each other.

**Table 1.** Summary of relationships between species richness, clade age, and diversification rates among randomly sampled clades. Results are based on PGLS analyses among clades. Each *r*^2^-value and *p*-value is the average from 10 replicates, each with 50 randomly selected clades (‘significant’ indicates the percentage of replicates with *p* < 0.05). Note that some clades within a replicate were deleted because of zero-length branches, including 4 in animals, 15 in plants, 24 in fungi, 66 in bacteria, 43 in archaeans, and 15 across life. Furthermore, 17 nested clades in archaeans were also excluded. We give the total sample size of clades below the name of each group. Clade ages are crown-group ages. Diversification rates (div. rate) are from the MS crown-group estimator with *ε* = 0.5. For the multiple regression analyses (clade age + div. rate), the *r*^2^-values are adjusted for multiple variables. Results based on OLS regression, stem-group clade ages, and alternative *ε*-values are given in electronic supplementary material, tables S1–S2, S4.

group	variables	*r*^2^	*p*	significant (%)
Animalia (*n* = 496)	richness versus clade age	0.2811	0.0723	90
	richness versus div. rate	0.1328	0.1925	50
	div. rate versus clade age	0.0491	0.1883	30
	richness versus clade age + div. rate	0.2986	0.0159	90
Plantae (*n* = 485)	richness versus clade age	0.2591	0.0334	80
	richness versus div. rate	0.1297	0.0702	40
	div. rate versus clade age	0.0590	0.1228	10
	richness versus clade age + div. rate	0.2661	0.0042	100
Fungi (*n* = 476)	richness versus clade age	0.3421	0.0226	80
	richness versus div. rate	0.0459	0.4385	20
	div. rate versus clade age	0.0325	0.2659	0
	richness versus clade age + div. rate	0.3340	0.0050	100
Bacteria (*n* = 434)	richness versus clade age	0.3673	0.0006	100
	richness versus div. rate	0.0321	0.4788	10
	div. rate versus clade age	0.0390	0.2158	0
	richness versus clade age + div. rate	0.4441	0.0005	100
Archaea (*n* = 440)	richness versus clade age	0.3748	0.0006	100
	richness versus div. rate	0.0185	0.5698	10
	div. rate versus clade age	0.0332	0.2736	0
	richness versus clade age + div. rate	0.3168	0.0360	90
across life (*n* = 485)	richness versus clade age	0.0908	0.1512	50
	richness versus div. rate	0.1188	0.1082	60
	div. rate versus clade age	0.0243	0.3510	10
	richness versus clade age + div. rate	0.0628	0.1537	40

We also tested the relationships between the two diversification-rate estimators (ClaDS2; MS estimator), between ClaDS2 diversification rates and richness, and between ClaDS2 diversification rates and clade crown-group ages. We also performed supplementary analyses in which we analysed relationships between speciation rates (*λ*_imean_ and *λ*_tipmean_, ClaDS2) and diversification rates (MS estimator), between speciation rates and species richness, and between speciation rates and clade crown-group ages (using PGLS). There was generally a strong, positive relationship between the two diversification rate estimators (see Results). Along with simulations (e.g. [10,21,22,24]) these results suggest that neither estimator is problematic.

We repeated these analyses across the 10 replicated sets of clades for each group and then summarized the mean *r*^2^-values and *p*-values across these replicates. The 500 clades sampled in each group are provided in electronic supplementary material, dataset S1, including the species richness, crown age, and diversification rates for each clade. R code for the statistical analyses are given in electronic supplementary material, dataset S3.

### (f) Effects of incomplete taxon sampling

These analyses implicitly assumed that taxon sampling is complete. Thus, if a sampled clade contained two species, we assumed that the clade would only contain two species if every species in the group was included in the tree. However, the overall trees were not fully complete for any group (although the number of sampled bacterial and archaean species was similar to the described numbers of species in these groups). In animals, plants, and fungi, the sampling was approximately 5–20% complete, based on the numbers of sampled species in each group given above, and the number of described species in Bánki *et al*. [1]. Nevertheless, this incomplete sampling might not determine our overall conclusions, since incomplete sampling can still reflect the relative richness of clades and the influence of diversification rates and clade ages on these relative richness patterns (e.g. relatively old clades with many sampled species will favour the clade-age hypothesis, whereas relatively young clades with many sampled species will favour the diversification-hypothesis). Furthermore, individual clades could be more complete at the species level than suggested by the overall sampling level (e.g. a selected genus could be well sampled at the species level even if most genera in the group were not included in the overall tree).

We therefore tested the influence of incomplete taxon sampling on relationships between richness, clade age and diversification rate. We used two approaches to do this. First, we repeated the main analyses in a group with a well-sampled phylogeny, and then randomly reduced the number of included species to 10% of the full tree, and then re-estimated relationships between richness, clade ages, and diversification rate. We used mammals as the focal group.

We downloaded 1000 time-calibrated trees from a posterior distribution of trees from a Bayesian analysis of mammals from VertLife (https://vertlife.org/phylosubsets/). This set of trees included 5911 species, covering approximately 90% of described species. We then made a consensus tree of these 1000 trees using the program TreeAnnotator from the BEAST package [29]. To summarize branch lengths across trees, the node heights in the consensus tree were set to ‘Common Ancestor Heights'. We first performed analyses on the full tree, randomly sampling clades. Next, we randomly selected 10% of the species to create a reduced tree, using the function ‘as.integer’ in the R package *base* [13] and ‘keep.tip’ in *ape*. We then randomly sampled clades from this reduced tree. The full and reduced trees are available in electronic supplementary material, dataset S4. The data and results are in electronic supplementary material, dataset S5.

As a second approach, we performed a limited set of analyses in which we used the described species richness of each sampled clade. We then compared these alternative results to those from our main analyses. To estimate richness, we obtained the number of described species for each clade that included two or more genera, using the CoL [1] and other sources (electronic supplementary material, dataset S6). Specifically, within a family, we summed the species richness of all sampled genera in that clade. If the clade included two or more families, we summed the described richness of the sampled families. We applied the same procedure to other higher taxa (e.g. orders). In contrast, if a sampled clade contained only species within a single genus, we only included the sampled species. Thus, we did not attempt to assign unsampled species to clades within genera (which would generally be very difficult without a complete species-level phylogeny). We used the revised species numbers to estimate diversification rates (MS crown-group estimator, *ε* = 0.5) and the relationships between species richness, clade ages, and diversification rates. Overall, this second approach should be an improvement over the main analyses. However, it was laborious and difficult to automate. Therefore, we applied this approach only for the first 40 clades for each group (excluding clades with zero-length branches). Furthermore, this approach still has important limitations (e.g. not accounting for incomplete species sampling within genera, or undescribed species). The data used and trees for PGLS are in electronic supplementary material, datasets S6 and S7, respectively.

We also performed analyses in which we analysed the set of 40 clades in four sets of 10 clades each, for a total of 24 sets across all six groups. We then tested whether *r*^2^ values from relationships between richness, clade ages and diversification rates were significantly different between this alternative approach and the main analyses (using paired, two-sample *t*-tests in *R*; all relationships were positive). Thus, we tested whether incomplete sampling likely biased the main results.

Overall, these two approaches (mammals, described richness) suggest that the main results should be robust to incomplete sampling (see Results). Similarly, our results should be robust to the presence of many undescribed species [15].

## 3. Results

**Figure 2.**
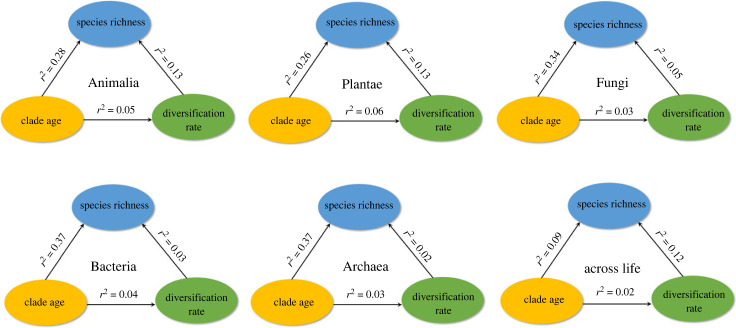
Pairwise relationships between species richness and clade age, species richness and diversification rate, and diversification rate and clade age. Species richness is log10-transformed. Clade age is the crown-group age. Diversification rate is inferred from the MS crown-group estimator with an *ε* of 0.5. For each group, we show the mean *r*^2^ from PGLS regression analyses. Full results are in table 1. We present species richness, clade age and diversification rate for each clade in electronic supplementary material, dataset S1.

The results for each replicate for each major group are given in electronic supplementary material, dataset S8. Within groups (animals, plants, fungi, bacteria, archaeans), there were generally significant positive relationships between richness and crown-group ages of clades using PGLS (80–100% of replicates; table 1; electronic supplementary material, table S1 and figure S1). Furthermore, clade age explained non-trivial variance in richness among clades (mean *r*^2^ = 0.26–0.37 depending on group; figure 2). By contrast, the relationship between clade age and species richness was weaker across life (*r*^2^ = 0.09), and only sometimes significant (50% of replicates; table 1; figure 2). Overall results were similar using stem-group ages and ordinary-least squares (OLS) regression (table 1; electronic supplementary material, table S1), but with somewhat weaker relationships using stem-group ages.

In contrast to the relationships between clade age and richness, those between richness and diversification rates (crown-group MS estimator, *ε* = 0.5) were generally weaker (*r*^2^ = 0.02–0.13) and not significant (10–60%; figure 2; table 1; electronic supplementary material, table S2). Multiple regression models (age + diversification) generally explained little additional variance in richness beyond clade age alone (table 1). However, when assuming *ε* = 0.9 for the MS estimator (electronic supplementary material, table S3), the richness-diversification relationship was often significant in animals (90%), plants (100%), and across life (90%) and stronger (mean *r*^2^ = 0.20–0.24). The relationship was generally non-significant and weak in fungi, bacteria and archaeans (mean *r*^2^ = 0.03–0.04; electronic supplementary material, table S3). Importantly, using the alternative MS estimator (*ε* = 0.9; electronic supplementary material, table S3) suggests that diversification rates explain the most variation in species richness among clades when clades are drawn from across the tree of life, whereas time and diversification rates explain similar variance in richness within plants and animals (animals: mean *r*^2^ for age = 0.28, diversification rates = 0.20; plants: age = 0.26, diversification = 0.24).

Diversification rates were not generally related to clade age (table 1; electronic supplementary material, table S4; figure 2). Therefore, weak relationships between diversification rates and richness seem unlikely to be explained by faster diversification rates in younger, species-poor clades, as previously found [7], and strong relationships seem unlikely to be artefactual. The relationships among species richness, clade age and diversification rates were similar when clades with diversification rates greater than 1 species/Myr were included (electronic supplementary material, tables S5–S7).

Using an alternative diversification-rate estimator (ClaDS2) gave estimated rates that were strongly and positively related to those using the crown-group MS estimator (electronic supplementary material, table S8). The relationship was least strong in animals, archaeans and across life (mean *r*^2^ = 0.73, 0.67 and 0.86, respectively) and stronger in other groups (mean *r*^2^ > 0.90). Relationships between diversification rates and richness among clades were generally similar between methods, with some exceptions. ClaDS revealed a generally significant relationship between diversification and richness in animals (mean *r*^2^ = 0.25) but not other groups (electronic supplementary material, table S8). Using the MS estimator, these relationships were typically significant in animals, plants and across life using an *ε* of 0.9 (electronic supplementary material, table S3) but not 0.5 (table 1). There were generally significant relationships between clade age and diversification rates (from ClaDS) in plants, fungi and across life (mean *r*^2^ = 0.25, 0.11 and 0.11), but not in other groups (electronic supplementary material, table S8); these relationships were typically non-significant using the MS estimator. Relationships between species richness, clade age and speciation rates (*λ*_imean_ and *λ*_tipmean_) from ClaDS2 were generally similar to those using diversification rates (electronic supplementary material, table S9).

Subsampling experiments with mammals (electronic supplementary material, table S10 and dataset S5) suggest that the relationships between species richness, clade age and diversification rates are similar between analyses with relatively complete species sampling (approx. 90%) and highly incomplete sampling (approx. 10%). Mean values of *r*^2^ were also similar, but were higher with incomplete sampling. Importantly, there was no evidence that the relative support for clade ages and diversification rates as explanations for richness patterns were an artefact of limited taxon sampling in the main analyses (table 1).

To further assess the impacts of incomplete sampling we analysed 40 clades for each group using numbers of described species for higher taxa (electronic supplementary material, table S11). For animals, we found stronger effects of clade age than diversification rates on richness (*r*^2^ = 0.45 versus *r*^2^ = 0.16; *n* = 40 clades; all relationships positive). There was a similar dichotomy for plants (clade age: *r*^2^ = 0.34, diversification rate: *r*^2^ = 0.11; *n* = 40), fungi (*r*^2^ = 0.49, *r*^2^ = 0.09), bacteria (*r*^2^ = 0.18, *r*^2^ = 0.12), archaeans (*r*^2^ = 0.38, *r*^2^ = 0.00) and across life (*r*^2^ = 0.30, *r*^2^ = 0.02). In general, these results parallel those from our main analyses (table 1). The main exception is across life, in which these alternative analyses favour clade age over diversification rates as the primary explanation for richness patterns. These alternative results bring the results from across life more in line with those from individual groups.

These alternative results were based on fewer clades than our main analyses. Therefore, we specifically compared our results from these alternative analyses to those from our main analyses for the same sampled clades (electronic supplementary material, table S11). Results were similar for plants, fungi, bacteria and archaeans. For animals, the relationships between species richness and both clade age and diversification rates were stronger using the main approach. However, the relative strength of the relationships remained similar, with clade ages explaining roughly twice as much variation in richness as diversification rates. Across life, there was a stronger relationship between clade age and richness in the alternative analyses than the main analyses. Overall, the main approach underestimated the relationship between clade age and richness about as often as it overestimated it (across these six groups). By contrast, the main approach overestimated the relationship between diversification rates and richness (for all groups but plants).

We also subdivided the data into sets of 10 clades to further test for biases in PGLS results between these approaches (electronic supplementary material, table S12). We found no significant difference in *r*^2^ values for richness and clade age (mean for main = 0.38, alternative = 0.46; *p* = 0.2994; *n* = 24) nor between richness and diversification rates (main = 0.25, alternative = 0.24; *p* = 0.8014; *n* = 24). In summary, these results suggest that incomplete taxon sampling does not strongly bias the relationships analysed here.

## 4. Discussion

The differences in species richness among clades encompass some of the most dramatic patterns of biodiversity, such as the dominance of angiosperms among plants and arthropods among animals. The general causes of these patterns (clade age versus diversification rates) have been highly uncertain given disagreements among previous studies. Furthermore, many past studies were potentially biased by only comparing clades of the same taxonomic rank. Here, we show that when we remove this potential source of bias, there is support for the clade-age hypothesis within all major groups of organisms analysed (animals, plants, fungi, bacteria, archaeans). This result contrasts strongly with some previous studies that focused on comparing clades of the same taxonomic rank [5,7]. We also found some support for the diversification-rate hypothesis, especially in animals, plants, and across life. However, the effect of diversification rates was generally weaker than that of clade age (except across life), and sensitive to the methods used to estimate rates (figure 2; table 1; electronic supplementary material, tables S3 and S8). Although our study has important limitations (e.g. incomplete species sampling, uncertainty about diversification-rate estimates), we performed many alternative analyses to show the general robustness of our conclusions. Our results should help resolve the conflicts among previous studies on this topic, and suggest that there may be general principles that explain diverse types of species richness patterns. We address the latter idea first.

### (a) Generalities in the causes of richness patterns

Combined with the results of other studies, our results suggest that there are broad generalities in the causes of diverse richness patterns. We found that patterns of richness among clades within groups are explained most frequently by variation in clade age, when clades are chosen randomly.

Based on large-scale reviews, spatial richness patterns among regions within clades are most often explained by when each region is successfully colonized by that clade, and not by spatial variation in diversification rates (e.g. [30,31]). Thus, regions that were successfully colonized earlier tend to have higher richness than those colonized more recently. This same explanation also applies to richness patterns along elevational and climatic gradients (review in [31]). Given that colonization events can potentially happen anywhere within a tree (from the youngest to the oldest clades), the relationship between colonization time and richness of regions may parallel the random selection of clades here, with older clades equivalent to older colonization events. However, simulations suggest that over longer time scales, diversification rates may become increasingly important in driving spatial richness patterns [32] (see also [33]). Here, diversification rates were only important in some groups and using some methods (table 1; electronic supplementary material, tables S3 and S8), and clade ages were the least important at the deepest time scale (across life; table 1).

A third type of richness pattern is trait-based richness: the number of species with each state or value of a character, such as a given diet, reproductive mode, or body size. In a review of case studies [34], the most species-rich states were generally those that evolved earliest, not the states with the highest diversification rates (although diversification rates were important in some studies). These character-state transitions can occur anywhere in a tree, again creating a parallel to the random selection of clades in clade-based richness patterns.

In summary, our results here and earlier studies suggest that clade age (and the timing of colonization and character-state transitions) may be the most frequent explanation for all three types of richness patterns. The major alternative is that differences in diversification rates explain these patterns instead. But differences in diversification rates may often need to be extreme to overturn the influence of time (i.e. a young clade must have a very high diversification rate to have as many species as an older clade). Furthermore, in phylogenies of extant species, the youngest clades will greatly outnumber the oldest clades. Thus, most randomly selected clades here were young and had few species (electronic supplementary material, figure S1). Similar patterns may arise for biogeographic and character-state transitions (i.e. all else being equal, transitions may occur more recently within a clade, when there may be more species extant).

There are also cases when diversification rates help explain these three types of richness patterns instead. Although time scale seems to matter for spatial richness, this is not so clear for clade-based patterns. For example, clade-age was still important within the oldest groups (archaeans, bacteria) and diversification rates were sometimes important in younger groups (animals, plants; table 1). On the other hand, the effect of clade age was not consistently significant across life (table 1), and richness patterns across life were often explained by diversification rates when using some alternative estimators (electronic supplementary material, table S3).

Finally, diversification rates and time are not the sole explanations for richness patterns. For example, variation in diversification rates among clades may be related to variation in traits among these clades, such as diet and habitat (review in [3]). For spatial richness patterns, various factors may help explain higher diversification rates in some regions, such as climate and geomorphology ([35,36]; but see [37] and others). There may be different levels of explanation for richness patterns. Thus, diversification rates can offer one level of explanation, but why those rates vary among clades is another level (note that ‘ecological limits’ represent another level of explanation, not a competing explanation relative to diversification rates or time [32]). However, it is crucial to test diversification rates and clade ages as competing explanations. The choice between these two will then guide analyses of further levels of explanation.

### (b) Resolving conflicts among previous clade-based studies

The causes of clade-based richness patterns have not been as widely studied as spatial richness patterns, yet there is considerable disagreement among the conclusions of previous broad-scale studies that have addressed this pattern. Here, we attempt to reconcile and explain these apparent conflicts.

McPeek & Brown [2] strongly favoured the clade-age hypothesis over the diversification-rate hypothesis, and tested both. Their study was primarily based on analyses of 163 species-level animal phylogenies. These trees were relatively young (median age = 7.5 Ma, upper 95% percentile = 30.3 Ma), which presumably made it difficult for differences in diversification rates to yield strong differences in richness. Furthermore, these analyses did not focus on comparing clades of the same rank. More puzzling is their comparison of insect and vertebrate orders, which showed a strong effect of clade ages and not diversification rates (using the MS estimator). However, later studies using more recent phylogenies (and the MS estimator) showed strong relationships between diversification rates and richness among insect orders [38] and major vertebrate clades [39]. Furthermore, Scholl & Wiens [7] tested these hypotheses on orders across animals and across life, and supported the diversification-rate hypothesis over the clade-age hypothesis in both cases.

Neither Rabosky *et al*. [5] nor Hedges *et al*. [6] directly tested the diversification-rate hypothesis, and their results on the clade-age hypothesis were in conflict. Rabosky *et al*. [5] compared taxa of the same rank (e.g. families) using stem-group ages in animals, plants, and fungi. They found no effect of clade ages on richness [5]. In this case, the lack of a relationship with clade ages may have been strongly influenced by only considering clades based on taxonomic ranks, and possibly the use of stem-group ages. Hedges *et al*. [6] supported the clade-age hypothesis based on named taxa (genera and families) of birds and mammals, but only when they used crown-group ages and not stem-group ages. We also supported the clade-age hypothesis in mammals, based on randomly selected clades and crown-group ages (electronic supplementary material, table S10).

Scholl & Wiens [7] analysed named clades across the tree of life, from families to phyla. They found little support for the clade-age hypothesis (using stem-group ages) and supported the diversification-rate hypothesis instead. Again, their results were likely biased against the clade-age hypothesis by focusing on named clades of the same rank (and stem-group ages). Here, we did not find a strong effect of using crown-group ages versus stem-group ages on the relationship between clade age and richness. Nevertheless, relationships between richness and clade age were generally weaker using stem-group ages (table 1, electronic supplementary material, table S1), which is consistent with these previous studies. In summary, few results of these previous studies directly contradict each other (or ours), either because key hypotheses were not tested, or different approaches were used to select clades and determine their ages.

As an aside, we repeatedly suggested that using named clades might bias comparison of clade ages (see also [3]), but without demonstrating this bias. We find here that standard deviations of clade ages for named clades (from [7]) are actually larger than from randomly selected clades (electronic supplementary material, table S13). This occurs because standard deviations in clade age generally increase with increasing mean clade ages (electronic supplementary material, table S13). Nevertheless, using named clades (i.e. families to kingdoms) does seem to bias mean clade ages to be older than those from randomly selected clades (electronic supplementary material, table S13). Randomly selected clades are predominantly younger, because in a time-calibrated phylogeny of extant species, there are far more younger clades than older clades (e.g. figure 1*a*).

### (c) Comparing diversification-rate estimators

The strong relationships found here between the two diversification-rate estimators (ClaDS, MS estimator; electronic supplementary material, table S8) have important implications. First, these results help validate previous inferences about clade-based richness patterns from the MS estimator [7]. Therefore, we did not repeat that study [7] using alternative methods: using ClaDS should yield similar results. Those authors [7] did not utilize a species-level phylogeny, but a family-level phylogeny instead (and trees among orders, classes, kingdoms and phyla). Thus, it was not possible to repeat their study using ClaDS or other methods that require a species-level tree. Second, our results support previous research [40] showing that rates from the MS estimator are correlated with those from other diversification-rate estimators for individual clades. Third, our study contrasts with an earlier one [41] that suggested that diversification-rate estimators based on the ages and richness of clades (like the MS estimator) were problematic, but without directly addressing their accuracy. That study [41] implicitly assumed that diversification-rate estimates from the clade-based MS estimator were uncorrelated with those from species-based estimators and with the true rates (otherwise, why would they be problematic?). Instead, our results here, and those of previous empirical and simulation studies, show that estimates from the MS estimator are correlated with both species-based estimators and the true rates. We also show that these clade-based diversification-rate estimates can be correlated with species richness and with estimates from other methods, despite considerable variation in the ages of the clades analysed. Finally, our results suggest that rate estimates from the MS estimator might be a reasonable proxy for those from ClaDS, when the only information available are clade ages and species richness. ClaDS requires a detailed, species-level, time-calibrated phylogeny. Further, we found that even when such a species-level phylogeny was available, ClaDS often failed to work on many individual clades.

## 5. Summary

Patterns of species richness among clades include some of the most striking patterns of biodiversity. Yet, the causes of these patterns have been controversial. We tested whether patterns of species richness among clades were related to the ages of these clades or to their diversification rates. By using randomly selected clades rather than clades of the same taxonomic rank, we show that richness is generally related to clade ages within each of the major groups of organisms, in contrast to some earlier studies. We also show that clade ages generally predict clade-based richness patterns more strongly than diversification rates. Nevertheless, diversification rates do help predict richness patterns in some cases, especially in animals, and clade ages do not consistently predict richness across all of life simultaneously. More broadly, our results help show that hypotheses based on time can be broadly important for explaining all three of the broad categories of richness patterns (spatial, clade-based and trait-based). Thus, our results suggest a common cause that frequently underlies these diverse types of biodiversity patterns.

## Data Availability

The data and code are provided in the electronic supplementary material [42].
